# Characteristic Properties of a Bamboo-Based Board Combined with Bamboo Veneers and Vacuum Heat-Treated Round Bamboo Sticks

**DOI:** 10.3390/polym14030560

**Published:** 2022-01-29

**Authors:** Yu-Hsuan Yang, Min-Jay Chung, Tung-Lin Wu, Chin-Hao Yeh, Teng-Chun Yang

**Affiliations:** 1 Department of Forestry, National Chung Hsing University, Taichung 402, Taiwan; babybear1314520@gmail.com (Y.-H.Y.); harrison19960219@gmail.com (C.-H.Y.); 2 Experimental Forest, College of Bio-Resources and Agriculture, National Taiwan University, Nantou 557, Taiwan; r90625001@ntu.edu.tw; 3 Department of Wood Science and Design, National Pingtung University of Science and Technology, Pingtung 912, Taiwan; tonywu@mail.npust.edu.tw

**Keywords:** vacuum heat treatment, round bamboo stick, rotary-cut bamboo veneer, dimension stability, flexural properties

## Abstract

In this study, a bamboo stick board with rotary-cut bamboo veneers was successfully fabricated. Additionally, vacuum heat (VH) treatment, which is a popular thermal modification method, was used to modify bamboo sticks. Therefore, the effects of different VH treatment temperatures on the dimensional stability and flexural properties of bamboo stick boards with and without bamboo veneers were investigated. For all boards, as the temperature increased to 220 °C, the thickness change rate and equilibrium moisture content decreased, and the flexural properties increased. The results exhibited that VH treatment improved the dimensional stability and flexural properties of the boards. Furthermore, the board with veneers had lower flexural properties and higher thickness swelling after water absorption than the board without veneers (BSB). The results indicated that bamboo veneer caused low flexural properties and high thickness swelling of the board compared to the BSB. However, the bamboo veneer played an aesthetic role in the appearance of the bamboo stick board.

## 1. Introduction

Bamboo is widely distributed and abundant across Asia. Additionally, bamboo has highly mechanical properties due to the longitudinal alignment of vascular bundles in its tissue structure and has a chemical composition of 60% cellulose with a high content of lignin [1,2,3]. Therefore, bamboo has generally been recognized as a very promising alternative raw material for the manufacturing of construction materials. Recently, academia and industry have developed bamboo-based materials, such as bamboo fiber-reinforced polymer composites [4,5,6], laminated bamboo lumber [7,8,9,10], reconstituted densified bamboo products [9,10,11,12,13], and unidirectional round bamboo stick boards [14]. Among these materials, reconstituted densified bamboo products have attracted investigation, since they are fabricated with high utilization of bamboo and possess highly mechanical properties [11]. However, Yu et al. [11] stated that a reconstituted densified product has a nonuniform density profile, since different cross-sectional dimensions of bamboo bundles are used as the raw materials. To improve the density profile, we developed a unidirectional bamboo-based board with various densities, made of round bamboo sticks, that shows uniform density profiles [14]. Additionally, the present study focused on thin bamboo veneer obtained by rotary cutting. There has been little information on the application of rotary-cut and thin bamboo veneers. Most previous studies explored the characteristic properties of the laminated bamboo veneer lumber manufactured from veneers made of bamboo bundles instead of rotary-cut veneers [15,16,17]. To further utilize the bamboo veneer, the bamboo stick board was combined with bamboo veneers in this study.

*Phyllostachys makinoi* (makino bamboo) and *Phyllostachys pubescens* (moso bamboo) are economically important and popular bamboo species in Taiwan. A previous study reported that makino bamboo has high flexural properties due to high holocellulose and α-cellulose contents [18]. Despite the excellent properties of makino bamboo, its application has been limited, due to a lower culm diameter and culm wall thickness than moso bamboo. Therefore, makino bamboo was processed into round bamboo sticks to prepare the bamboo stick board (BSB) in this study. Moso bamboo has been explored by many studies, since it is a widely harvested bamboo species commercially in Asia and has interesting chemical, anatomical, and physico-mechanical properties [2,3,19,20,21,22,23]. Additionally, the size of each part of moso bamboo is greater than that of makino bamboo, especially the culm wall thickness. In this study, rotary-cut bamboo veneers obtained from moso bamboo were applied to BSB. However, bamboo is a hydrophilic lignocellulosic material, due to its chemical components, such as cellulose, hemicellulose, lignin, and extracts. Bamboo is known to have high hygroscopicity and low thermal stability, resulting in its dimensional instability and biological degradation. To improve these disadvantages, a thermal modification method with eco-friendly and nontoxic byproducts has received more attention. Generally, heat treatment is used under different media (water, steam, nitrogen, and oil) at a temperature of 150–260 °C. In addition to media and temperature, the heating rate and duration also affect the properties of wood and bamboo [24,25,26,27,28,29]. Vacuum heat treatment (VH) is a popular thermal modification method that is suitable for biomass pyrolysis, carbonization, and heat treatment of lignocellulosic materials [30,31,32,33,34,35]. This treatment mainly replaces oxygen with a partial vacuum, and materials are heated by forced convection. Allegretti et al. [30] conducted VH treatment to modify two wood species, spruce (*Picea abies* Karst.) and fir (*Abies alba* Mill.), under various conditions (temperature, duration, and pressure) and determine their properties. Candelier et al. [31] explored the influence of vacuum or nitrogen on the chemical modification that occurred for wood during treatment. Lin et al. [32] investigated the change in chemical structure and composition of two wood species (poplar and fir) during heat treatment in a semi-industrial scale reactor in vacuum. Jebrane et al. [34] and Pockrandt et al. [35] evaluated the influence of steam heat treatment and VH treatment on the chemical, physical, and mechanical properties of woods. Todaro et al. [36] measured the thermal properties (thermal conductivity and diffusivity) and physical properties (porosity, mass loss, and surface color) of VH-treated black poplar (*Populus nigra* L.) wood. Furthermore, Lin et al. [32] reviewed the several advantages of heat treatment with vacuum for wood: (1) efficient drying; (2) decreasing odor of heat-treated wood; (3) greater color homogeneity on the surface of wood; (4) efficient reduction of hygroscopicity of wood heated under vacuum compared to other media; (5) high reactivity of wood thermal degradation; (6) easier and cheaper management for produced volatile wastes; and (7) treatment with reduced energy consumption and duration. According to these studies, VH treatment allowed for the removal of volatile degradation products in wood, limiting acidic degradation of polysaccharides. Additionally, VH treatment produced wood products with a higher durability against decaying fungi and a higher retention ratio of mechanical properties compared to other heat treatments [35]. To the best of our knowledge, a BSB with veneers (VBSB) has not been reported in the literature. Furthermore, there is little information on the investigations into the characteristic properties of a VBSB with VH-treated bamboo sticks. Accordingly, the main objective of the present study was to evaluate the 24 h soaking dimension ability and physical and flexural properties of BSB and VBSB with bamboo sticks treated at different treatment temperatures under vacuum. Furthermore, a comparison of properties of the board with and without bamboo veneers was conducted.

## 2. Materials and Methods

### 2.1. Materials

**Figure 1:**
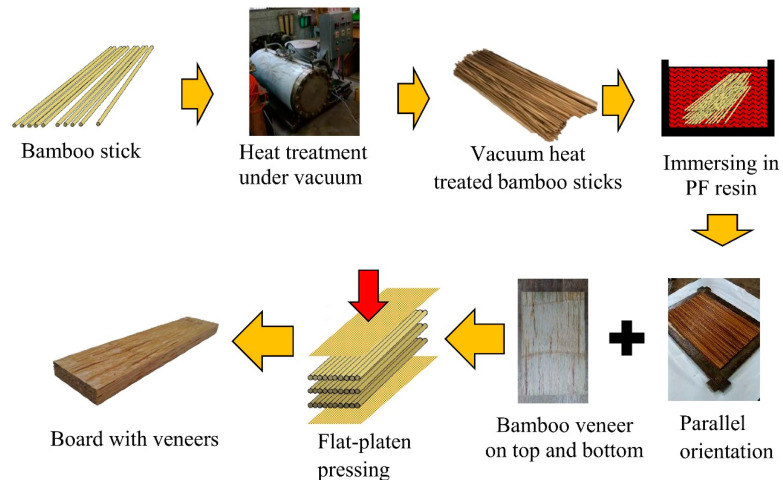
Manufacturing process of a bamboo stick board with veneers.

As shown in Figure 1, round makino bamboo sticks of 297 mm × 3 mm (length × diameter) were manufactured from peeled bamboo (3 years old; *Phyllostachys makinoi*) strips without the outer and inner layers. Bamboo veneers of 297 mm × 185 mm × 0.5 mm (length × width × thickness) were obtained from moso bamboo culms (3 years old; *Phyllostachys pubescens*) by a rotary cutter. Bamboo sticks and bamboo veneers were obtained from a local factory in Nan-Tou County, Taiwan. The adhesive was a liquid phenol formaldehyde (PF) resin obtained from Kuen Bong Chemical Industry Co. (Ilan, Taiwan). The viscosity of PF resin was 125 ± 25 cps, the pH value was 8.1 ± 0.4, the solid content was 63.5 ± 2.5%, and the specific gravity was 1.22 ± 0.02.

### 2.2. Heat Treatment under Vacuum

The bamboo sticks were heated under vacuum in a laboratory-scale treatment reactor (Figure 1). It consisted of a stainless-steel vessel containing heating plates and connected to a pressure sensor, a pressure valve, and a vacuum pump. The bamboo sticks were first oven-dried at 105 °C for 12 h. Before heat treatment, the pressure in the reactor under vacuum was fixed at 0.25 atm for 30 min. The heating plates were further heated from 105 °C to the desired temperature (180, 200, and 220 °C) at a heating rate of 3 °C/min. Subsequently, the treatments maintained the desired temperature for 2 h, and the bamboo sticks were cooled to room temperature at 5 °C/min. Before and after VH treatment, the bamboo sticks were stored in a conditioned room at 25 ± 1 °C and 65 ± 5% relative humidity (RH).

### 2.3. Manufacturing Process of the Boards

**Figure 2:**
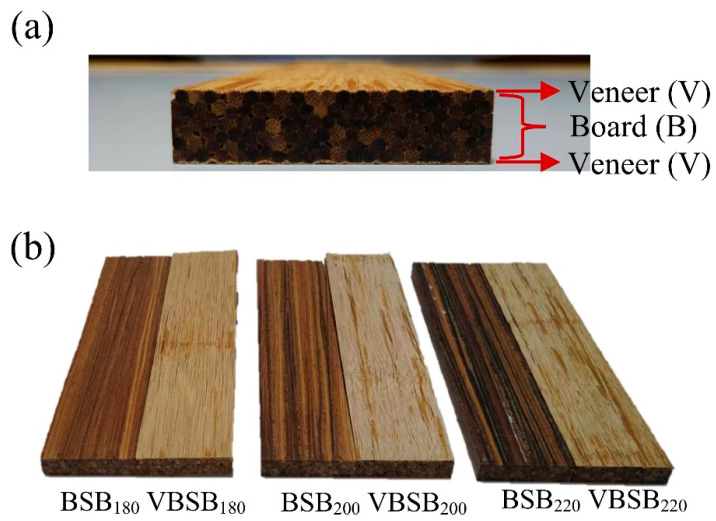
(**a**) Cross-section of a bamboo stick board with veneers; (**b**) appearance of bamboo stick boards (BSBs) and bamboo stick boards with veneers (VBSBs).

Figure 1 illustrates the flat-platen pressing process that was applied to manufacture the bamboo stick board with veneers. The oven-dried bamboo sticks were immersed in PF resin, and the loading of the PF resin was 10 wt%. For the bamboo stick board without veneers (BSB), the bamboo sticks with PF resin were parallelly assembled in the mold with dimensions of 300 mm × 200 mm × 12 mm (length × width × thickness). Subsequently, a two-step pressing process was used to produce the boards as follows: (1) hot pressing at a temperature of 150 °C and pressure of 90 kgf/cm^2^ for 10 min and (2) finishing by cold pressing for 10 min. The expected density of all boards was 900 kg/m^3^. For the bamboo stick board with veneers (VBSB), oven-dried and untreated bamboo veneer was placed on the top and bottom of the parallel-assembled bamboo sticks (Figure 2a). To obtain the lowest difference in density between the BSB and VBSB, the amount of bamboo sticks per volume was lower for the VBSB than for the BSB. The boards with and without veneers were denoted as BSB_X_ and VBSB_X_, respectively, where X is the heat treatment temperature for the bamboo sticks (Figure 2b). Prior to testing, the boards were stored in a conditioned room at 25 ± 1 °C and 65 ± 5% RH for 2 weeks.

### 2.4. Characteristic Properties

The density, equilibrium moisture content (EMC), water absorption (WA), and thickness swelling (TS) of all boards (sample size: 50 mm × 50 mm × 12 mm) were determined according to test procedure ASTM D1037-12. According to ASTM D790-17, the modulus of rupture (MOR) and modulus of elasticity (MOE)—using a three-point static flexural test with a support span of 16 times the depth of the board and a crosshead speed of 10 mm/min—were assessed (sample size: 230 mm × 50 mm × 12 mm). Thickness change rate (TCR) was calculated according to the following equation: TCR (%) = (*T*_a_ − 12)/12 × 100, where *T*_a_ is the thickness after being in a controlled environment (mm).

### 2.5. Analysis of Variance

The statistical analysis was performed by Scheffe’s test using the Statistical Analysis System. A significance level of 5% was considered for all of the analyses.

## 3. Results and Discussion

### 3.1. Physical Properties

**Figure 3:**
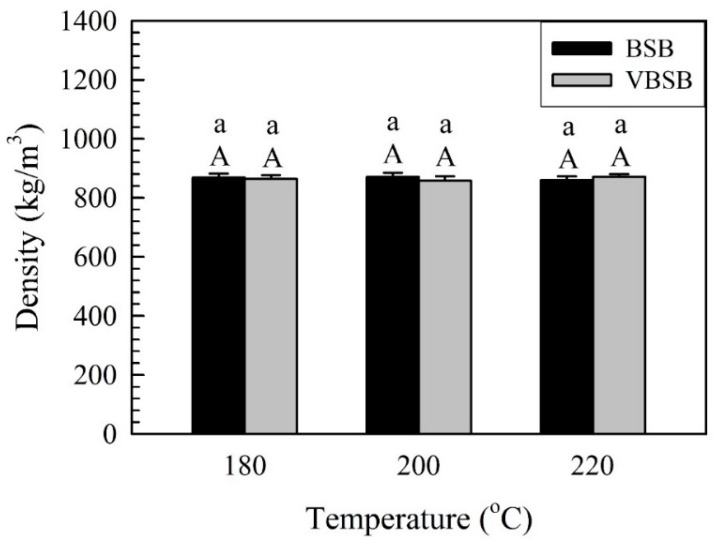
Density of bamboo stick boards with and without veneers. Bars with capital letters indicate significant differences between various temperatures. Bars with lowercase letters indicate significant differences between boards with and without veneers.

The density of the bamboo stick boards with and without veneers is illustrated in Figure 3.

**Figure 4:**
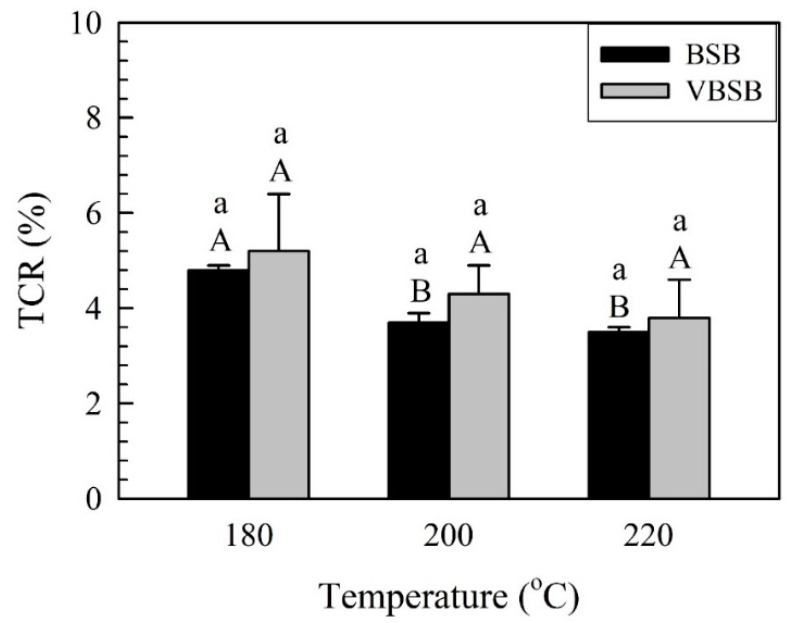
TCR of bamboo stick boards with and without veneers. Bars with capital letters indicate significant differences between various temperatures. Bars with lowercase letters indicate significant differences between boards with and without veneers.

Generally, density is one factor that directly impacts the flexural properties of a material. The density ranged from 858 to 871 kg/m^3^ for all samples, and there was not a significant difference in the density of the samples observed. Additionally, the densities of all samples were less than the expected density (900 kg/m^3^). This is related to the increased thickness of the board caused by spring back after flat-platen pressing and thickness swelling after being in a controlled environment. As shown in Figure 4, for the board without veneers (BSB), the thickness change rate (TCR) of BSB_180_ was 4.8%, and the rate decreased to 3.7% and 3.5% for BSB_200_ and BSB_220_, respectively.

**Figure 5:**
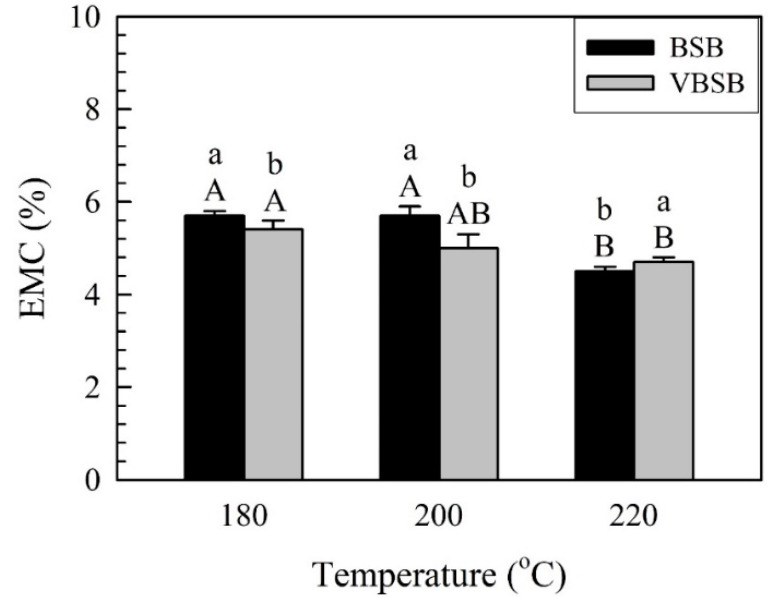
EMC of bamboo stick boards with and without veneers. Bars with capital letters indicate significant differences between various temperatures. Bars with lowercase letters indicate significant differences between boards with and without veneers.

This implies that the TCR decreased with increasing treatment temperature. Similar to the TCR of BSB, the TCR of the board with veneers (VBSB) decreased from 5.2% (VBSB_180_) to 3.8% (VBSB_220_) as the temperature increased to 220 °C. However, there were no significant differences between the TCRs of all VBSBs, according to the statistical analysis. Regardless of whether there are veneers, the TCR is affected by the spring back and thickness swelling of vacuum heat-treated bamboo sticks. At a given treatment temperature, the average TCR of the VBSB was higher than that of the BSB, but the statistical analysis resulted in an insignificant difference between the TCRs of the boards with and without veneers. These results indicate that the reduction in the TCR for all samples is attributed to the VH-treated bamboo stick having a lower spring back after flat-platen pressing and less thickness swelling after being in a controlled environment. Furthermore, the equilibrium moisture content (EMC) value can be used to indirectly determine the influence of VH-treated bamboo sticks on the thickness swelling after being in a controlled environment. As shown in Figure 5, the average EMC was 5.7%, 5.7%, and 4.5% for BSB_180_, BSB_200_, and BSB_220_, while it was 5.4%, 5.0%, and 4.7% for VBSB_180_, VBSB_200_, and VBSB_220_, respectively.

These results indicate that the EMC of all samples significantly decreased when the treatment temperature increased to 220 °C, which indirectly indicates that the boards with 220 °C-treated bamboo sticks had the lowest thickness swelling after being in a controlled environment. The EMC was mainly influenced by the hygroscopicity of the bamboo sticks in all samples. Therefore, heat treatment could result in moisture-sensitive bamboo sticks becoming hydrophobic to decrease the EMC of the samples, since hemicelluloses are removed and the cellulosic crystallinity increases [26,37]. Borrega and Kärenlampi [38] reported that heat treatment reduced the hygroscopicity of wood by the hornification effect, which is the hydrogen bonding between adjacent carbohydrate elements. Additionally, VBSB showed a lower EMC than BSB when the temperature was less than 200 °C. This may be related to the lower amount of bamboo stick in the VBSB. As described above, the lower amount of bamboo stick was used to manufacture the VBSB in order to obtain the lowest difference in density between the boards with and without veneers. Conversely, the EMC of VBSB_220_ was higher than that of BSB_220_. Previous studies reported that considerable degradation of non-crystalline cellulose and hemicellulose was observed in bamboo when the temperature increased to 220 °C, resulting in a significant reduction in the EMC of bamboo treated at 220 °C [14,27,39,40]. Therefore, the hygroscopicity of the bamboo veneers may have more of an effect on the EMC of the VBSB than that of the 220 °C-treated bamboo sticks, resulting in the higher EMC in the VBSB_220_ compared to BSB_220_.

### 3.2. Flexural Properties

**Figure 6:**
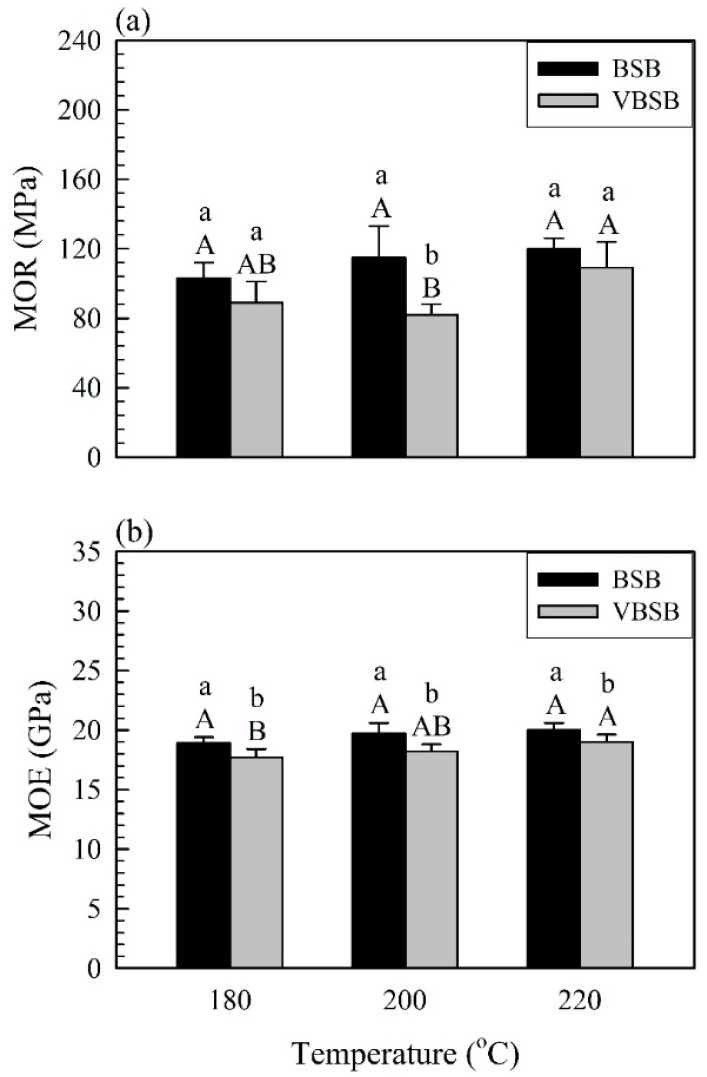
MOR (**a**) and MOE (**b**) of bamboo stick boards with and without veneers. Bars with capital letters indicate significant differences between various temperatures. Bars with lowercase letters indicate significant differences between boards with and without veneers.

In this study, flexural properties are the predominant factor in determining whether materials are applicable for structural applications. The MOR and MOE for BSB and VBSB with heated bamboo sticks are shown in Figure 6.

**Figure 7:**
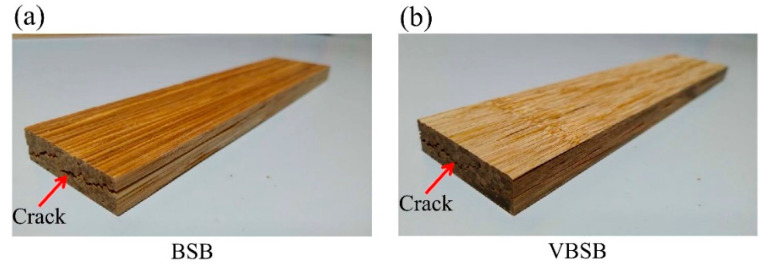
Appearance of the BSB (**a**) and VBSB (**b**) after the flexural test.

The average flexural properties (MOR and MOE) of the BSBs increased as the treatment temperature increased to 220 °C; however, there were no significant differences among the samples. The BSB with the heat-treated bamboo sticks exhibited flexural properties with MOR and MOE in the ranges of 103–120 MPa and 18.9–20.0 GPa, respectively. In a previous study by Yang and Lee [14], BSBs with higher density (1000 kg/m^3^) and various heat-treated bamboo sticks were fabricated, and the MOR and MOE were reported to significantly decrease when bamboo sticks were thermally treated at 220 °C. Decreased mechanical properties of bamboo are attributed to the depolymerization of hemicellulose and cellulose and the separation of the hemicellulose–lignin copolymer caused by heat treatment [27,41,42,43]. Interestingly, as the treatment temperature increased, a reduction in the MOR of BSB with a lower density (900 kg/m^3^) was not observed in this study. This may be related to the failure mode after the flexural test. Figure 7 shows the appearance of the failure modes of the BSB and VBSB.

The main shear crack for all samples propagated in the middle of the board thickness, which eventually caused bonding delamination along stick-adhesive interfaces. Accordingly, the increase in the MOR of BSB with bamboo sticks treated at higher temperature may be dependent on the improvement of the bonding or shear strength between the stick interfaces. Similarly, the MOR of VBSB increased from 89 (VBSB_180_) to 109 MPa when the heat treatment temperature reached 220 °C. Similar to the MOR results, heat treatment resulted in the VBSB having an increase in MOE up to 220 °C, and, thus, the VBSB_220_ had the highest MOE (19 GPa) among all VBSBs (Figure 6b). These results implied that heat treatment at higher temperatures (>200 °C) may improve the flexural properties of the board with a lower density (<900 kg/m^3^). At a given treatment temperature, the MOR and MOE of the VBSB decreased by 9.2–28.7% and 5.3–7.6%, respectively. This phenomenon resulted from the lower amount of bamboo stick used to manufacture the VBSB in order to obtain the expected density, causing the flexural properties to be lower than those of the BSB. The results indicate that the MOR and MOE of the boards were seriously influenced by the heat-treated bamboo sticks and veneers. Although the veneer resulted in reduced flexural properties, its bamboo texture on the upper and lower surfaces of the board can increase aesthetics (Figure 2).

### 3.3. Dimensional Stability after 24 h of Soaking

**Figure 8:**
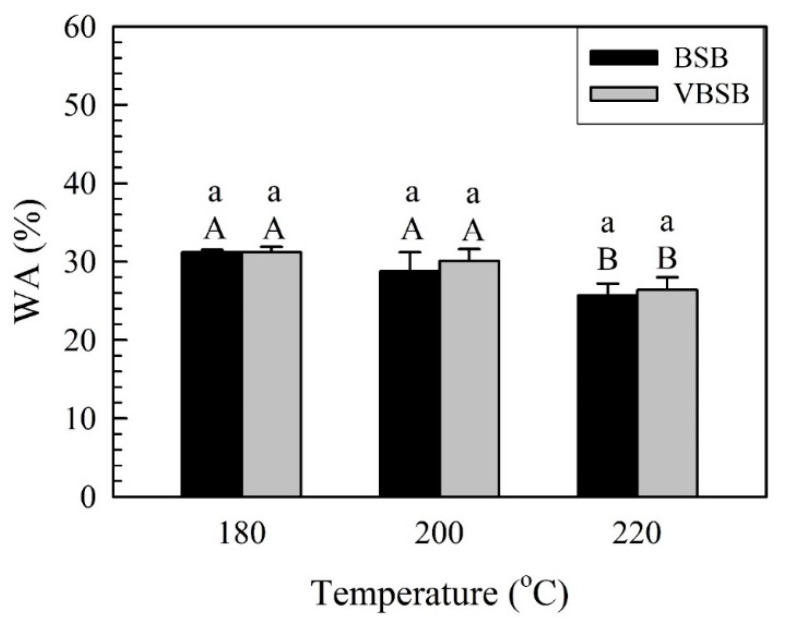
WA of bamboo stick boards with and without veneers. Bars with capital letters indicate significant differences between various temperatures. Bars with lowercase letters indicate significant differences between boards with and without veneers.

As estimated above, the EMC of all boards significantly decreased when bamboo sticks were heated at higher temperatures, due to a reduction in the hygroscopicity of bamboo. In this study, the dimensional stability of BSB and VBSB was estimated by water absorption (WA) and thickness swelling (TS) during water immersion. Figure 8 presents the WA of BSB and VBSB with bamboo sticks heated at various treatment temperatures.

**Figure 9:**
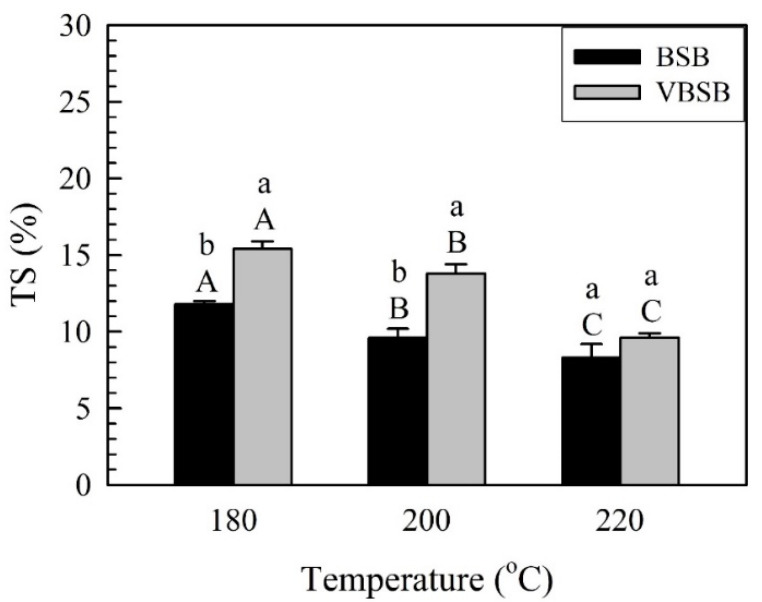
TS of bamboo stick boards with and without veneers. Bars with capital letters indicate significant differences between various temperatures. Bars with lowercase letters indicate significant differences between boards with and without veneers.

For BSB, the WA significantly decreased from 31.2% (BSB_180_) to 25.7% (BSB_220_) as the treatment temperature increased. Additionally, the WA of VBSB had the same decreasing trend as the temperature increased to 220 °C. The WA of VBSB_220_ exhibited the lowest WA (26.4%) among all VBSBs. The WA behavior of raw bamboo materials is related to hydrogen bonds that are formed from the linkage of water molecules and free hydroxyl groups in the cell wall of bamboo and water penetrating parenchyma cells and vessels [44,45]. For further comparison of the BSB and VBSB at a given treatment temperature, the average WA of the VBSB was higher than that of the BSB; however, the treatment temperature did not exhibit a significant impact on the WA of the boards with and without veneers, according to the statistical analysis. Figure 9 depicts a comparison of the TSs of bamboo stick boards with and without veneers.

The TS of the BSB with 220 °C-treated bamboo sticks significantly decreased from 11.8% (BSB_180_) to 8.3%, while this value of the VBSB showed a significant decrease to 9.6% at 220 °C. The bamboo sticks heated at higher temperatures are shown to cause a significant decrease in the TS of BSB and VBSB. These results mainly correspond to the further decrease in the hygroscopicity of bamboo sticks at higher temperatures due to the hornification effect and hemicellulose hydrolysis [27,38,46,47]. Therefore, the board with bamboo sticks heated at higher temperature had lower WA and TS values, which indicates better dimensional stability during water immersion. Furthermore, the average TS increased by 30.5% for VBSB_180_, 43.8% for VBSB_200_, and 15.7% for VBSB_220_ compared to each BSB. These phenomena have been mainly attributed to the fact that an increase in TS of untreated bamboo veneers results in an increased total TS of the VBSB. However, the board with 220 °C-treated bamboo sticks had the lowest difference in TS between BSB and VBSB.

## 4. Conclusions

The aim of this study was to investigate the flexural properties and dimensional stability during water immersion of boards with bamboo sticks (BSBs) treated by vacuum heat (VH) modification at various temperatures. Additionally, a comparison of the various properties of BSB and BSB with bamboo veneers (VBSB) was performed. For individual boards, the results indicated that most of the physical (TCR and EMC) and dimensional (WA and TS) properties significantly decreased as the treatment temperature increased to 220 °C, while flexural properties (MOR and MOE) showed an increasing trend as the treatment temperature increased. A comparison of the board with and without bamboo veneers showed that the VBSB had lower flexural properties and higher TS after 24 h of soaking compared to the BSB. However, the natural beauty of the bamboo veneer increased the aesthetic appearance of the BSB. Accordingly, these results indicated that VH treatment and bamboo veneers had positive and negative effects on the properties of BSB. The VBSB with VH-treated bamboo sticks may have the potential to be used in specific applications, such as decking and fencing. However, the VBSB has sufficient mechanical performance, but the water absorption and thickness swelling are still so high as to be limited for outdoor applications. Further studies are ongoing to investigate the workability and the optimal manufacturing process for improving the physical and mechanical properties of the VBSB, which could be used as a sustainable and eco-friendly green construction material in industry.

## Data Availability

The data presented in this study are available on request from the corresponding author.
